# The correlation and auxiliary diagnostic value of gut microbiota and serological indicators with Kawasaki disease complicated by coronary artery lesions

**DOI:** 10.3389/fcimb.2026.1865661

**Published:** 2026-09-03

**Authors:** Shanshan Yang, Yan Ding, Fan Liu, Shasha Wang, Jing Chen, Yali Wu

**Affiliations:** Department of Immunology and Rheumatology, Wuhan Children’s Hospital (Wuhan Maternal and Child Healthcare Hospital), Tongji Medical College, Huazhong University of Science & Technology, Wuhan, Hubei, China

**Keywords:** association, coronary artery lesions, gut microbiota, Kawasaki disease, serological indicators

## Abstract

**Objective:**

To compare the differences in gut microbiota diversity and serological parameters between children with Kawasaki disease (KD) complicated by coronary artery lesions (CAL) and those without CAL, and their association and auxiliary diagnostic value.

**Methods:**

A retrospective study was conducted on 57 KD children with CAL (CAL group) and 102 KD children without CAL (control group) diagnosed and treated in our hospital from January 2022 to December 2023. The gut microbiota diversity (Ace index, Shannon index), serum inflammatory markers and CD4^+^/CD8^+^ were compared between the two groups. Pearson correlation analysis was used to explore the association between gut microbiota and serological parameters. Logistic regression analysis was employed to identify independent influencing factors. Receiver operating characteristic (ROC) curve analysis was performed to evaluate the indicators.

**Results:**

Compared with the control group, the levels of serum CRP, TNF-α, IFN-γ, IL-2, IL-4, IL-6, IL-10, and CD4^+/^CD8^+^ ratio in the CAL group were significantly elevated, while the Ace index (an α-diversity indicator reflecting gut microbiota species richness) was significantly decreased (*P < 0.05*). Pearson correlation analysis showed that the Ace index was negatively correlated with serum CRP, TNF-α, IFN-γ, IL-2, IL-4, IL-6, IL-10, and CD4^+/^CD8^+^ ratio (*P < 0.05*). Multivariate Logistic regression analysis (with variance inflation factor VIF<5 for all variables, indicating no significant multicollinearity) demonstrated that the Ace index, pro-inflammatory factors (CRP, TNF-α, IFN-γ, IL-6, IL-4), CD4^+/^CD8^+^ ratio and the anti-inflammatory cytokine IL-10 were independently associated with CAL (*P < 0.05*). ROC curve analysis showed that the area under the curve (AUC) for the combined detection was 0.911 (95% CI: 0.860–0.962), with a specificity of 81.37% and a sensitivity of 87.72%, and the efficacy was superior to any single indicator.

**Conclusion:**

The Ace index, pro-inflammatory factors (CRP, TNF-α, IFN-γ, IL-6, IL-4), and CD4^+/^CD8^+^ ratio and IL-10 were independently associated with CAL. The combined detection of the above indicators demonstrated good efficacy for KD complicated by CAL and might provide clinical reference for early identification of high-risk children.

## 1 Introduction

Kawasaki disease (KD) is an acute self-limiting systemic vasculitis affecting small to medium-sized arteries that mainly affects children under 5 years old. Its clinical symptoms include persistent high fever, peripheral vascular changes (such as erythema, edema, and desquamation of the palms and soles), polymorphous rash, bilateral conjunctival injection, alterations in the lips and oral cavity, and acute non-purulent cervical lymphadenopathy (Rife and Gedalia, 2020). Although the exact cause of KD is not yet fully understood, it is currently widely believed that its pathogenesis is rather complex, involving multiple aspects such as genetic susceptibility, infectious agents, immune system dysfunction, and environmental influences (Sakurai, 2019; Seki and Minami, 2022). In China, the incidence of KD has shown a significant upward trend in recent years. According to the monitoring data in some areas, its incidence has reached 70–100 cases per 100,000 children under the age of 5, and it has become one of the main causes of acquired heart disease in children (Lin et al., 2022). KD can involve systemic blood vessels. When not treated in time, it may lead to the occurrence of coronary artery lesions (CAL), and even cause permanent structural damage to the coronary arteries, affecting the prognosis of children (Kuo, 2023). CAL is one of the common complication of KD. Data indicate that approximately 20% of patients with KD without standardized treatment will develop CAL, and after standardized treatment, this incidence can be reduced to 10% (Zhang et al., 2021). CAL not only cause acute heart problems, but also poses long-term risks, including coronary artery stenosis, thromboembolism, and premature arteriosclerosis, thereby elevating the risk of cardiovascular events (Selmek and Harding, 2024). Therefore, in-depth investigation into the pathogenesis and management of KD complicated with CAL is imperative for assessing the condition in pediatric patients and improving their prognosis.

The gut microbiota, as the largest microbial community in the human body, plays a critical role in maintaining host immune homeostasis and metabolic balance. The dysbiosis of intestinal microbiota is not only associated with a range of gastrointestinal diseases, but has also been found to be closely linked to inflammatory bowel disease (Lavelle and Sokol, 2020), cancer (Gori et al., 2019), cardiovascular disease (An et al., 2024), and other diseases. The main pathological change of KD is vasculitis, which may be associated with the abnormal activation of immune system. Changes in gut microbiota may affect the function of the gut immune system, thereby triggering systemic immune disorders (Kawamoto et al., 2023). Research has indicated that the number of beneficial bacteria such as *Bifidobacterium* and *Lactobacillus* in children with KD was decreased, while the number of pathogenic bacteria such as Clostridium was increased. The dysbiosis of intestinal flora may influence the occurrence and development of the diseases through multiple mechanisms (Shen et al., 2020). At the same time, dysbiosis of gut microbiota may also affect changes in serological indicators, including elevated levels of white blood cells, neutrophils, and C-reactive protein (CRP). These alterations may also be associated with the pathogenesis and progression of KD and its complications (Wang F. et al., 2023). Previous research has identified that various serum factors such as interleukin-2 (IL-2), IL-6, IL-10, and tumor necrosis factor-α (TNF-α) may play a role in the pathogenesis of KD and the associated coronary artery damage (An et al., 2021). Nonetheless, the diagnostic sensitivity and specificity of individual serological indicators for KD with concurrent CAL remain inadequate. Consequently, it is clinically significant to conduct a systematic comparison of the intestinal microbiota and serological indicators between children with concurrent and non-concurrent CAL, and to investigate the discriminatory efficacy of their combined application.

Based on this, the present study aims to examine the correlation between KD complicated with CAL and alterations in the intestinal microbiota by analyzing relevant clinical data, and to explore whether it can assist in evaluating the risk of KD complicated with CAL, providing reference for clinical decision-making.

## 2 Materials and methods

### 2.1 Study objects

A retrospective selection was conducted involving 57 children diagnosed with KD complicated with CAL who were admitted to our hospital between January 2022 and December 2023, and they were classified as the CAL group. Inclusion criteria: (1) Patients met the diagnostic criteria revised by the American Heart Association (AHA) guidelines in 2024 for KD (Jone et al., 2024) and were confirmed through Doppler ultrasound examination. (2) Course of disease < 10 days. (3) Patients had not received treatment with aspirin, corticosteroids, immunosuppressants, or immunoglobulin before admission. (4) The patient was initially diagnosed with KD. (5) Patients had complete laboratory tests, echocardiography, and clinical data. Exclusion criteria: (1) Patients with concomitant malignant tumors; (2) Patients with hematological, autoimmune, or infectious diseases; (3) Patients with a history of chronic gastrointestinal diseases and bacteremia; (4) The patient was complicated with hemorrhagic rash, infectious conjunctivitis, purulent lymphadenitis and other infectious diseases, which might cover the typical clinical manifestations of KD and interfere with the diagnosis; (5) Cases that were transferred to another hospital or lost to follow-up during the study period. 102 children with KD who were treated in our hospital during the same period and did not have concomitant CAL were selected as the control group. Inclusion criteria: (1) Patients met the diagnostic criteria revised by the American Heart Association (AHA) guidelines in 2024 for KD (Jone et al., 2024). (2) The disease course was less than 10 days and it was in the acute stage of KD (within 10 days of onset, and before receiving intravenous immunoglobulin (IVIG) or high-dose aspirin treatment) (3) All fecal and blood samples of the children were collected before IVIG or high-dose aspirin treatment, and the above drugs were not used before admission. (4) The patient was initially diagnosed with KD. (5) Patients had complete laboratory tests, echocardiography, and clinical data. Exclusion criteria: (1) Patients with concomitant malignant tumors; (2) Patients with hematological, autoimmune, or infectious diseases; (3) Patients with a history of chronic gastrointestinal diseases and bacteremia; (4) Patients with concurrent symptoms that could be easily confused with KD, such as hemorrhagic rash, infectious conjunctivitis and suppurative lymphadenitis; (5) Cases that were transferred to another hospital or lost to follow-up during the study period. The average age of the children was 2.50 ± 0.70 years, with 72 males and 30 females. Typical clinical symptoms: 100 cases of fever, 55 cases of rash, 76 cases of non-purulent cervical lymphadenopathy, and 74 cases of strawberry tongue. There were no significant differences in the general clinical data of the two groups of children (P > 0.05). According to previous studies (Do et al., 2020), the children were divided into two groups based on whether they developed resistance to IVIG: the IVIG-sensitive group (whose body temperature returned to normal within 48 hours after IVIG administration) and the IVIG-resistant group (whose body temperature remained > 38.5°C 48 hours after IVIG administration). Currently, there is no unified standard for classifying KD as mild, moderate, or severe. Moreover, it has been confirmed that IVIG resistance is associated with a higher risk of CAL (Do et al., 2020). Therefore, IVIG sensitivity/resistance was used as the basis for severity grouping. Those with IVIG sensitivity were combined into the mild/moderate group, while those with IVIG resistance were defined as the severe group.

This study is a retrospective analysis involving human samples and clinical data. It has been approved by the Ethics Committee of Wuhan Children’s Hospital (ethics approval number: 2021R1172-E01). All procedures were carried out in accordance with the approved protocol by the hospital’s ethics committee. For underage participants, written informed consent was obtained from their legal guardians or close relatives.

### 2.2 Evaluation of CAL

The Doppler ultrasound examinations were conducted using the Philips IE33 color Doppler echocardiography system, with an S8–3 pediatric-specific heart probe being employed. All the examinations were conducted by the fixed senior physicians of the Children’s Heart Center of our hospital (using the single-person dual-assessment process: that is, the same child was independently evaluated by two different physicians). During the examination, the child was placed in a supine position and kept in a quiet state. The probe frequencies were set at 2.25 MHz and 5.0 MHz.

Coronary circulation function was evaluated using transthoracic Doppler ultrasound, and a routine cardiac scan was conducted on each patient. Color Doppler ultrasound imaging technology was utilized to assess the diameter of the coronary artery and its branches, left ventricular systolic function, pericardial effusion, and other related manifestations. Color Doppler Flow Imaging (CDFI) revealed slow blood flow signals within the coronary arteries, which could manifest as eddies. The left ventricular reserve function of children with KD was evaluated using Doppler ultrasound technology. All the children were re-examined in the 2-3rd week of the disease course and at the 8th week after the onset to monitor the progression of CAL.

The severity and characteristics of CAL were assessed based on echocardiographic findings. The coronary artery diameter Z-score was employed to quantify coronary artery dilatation or aneurysm formation, which is considered as coronary artery abnormalities (CAA). The Z-score was utilized to evaluate the severity of CAL. A Z-score greater than 2 was indicative of the presence of CAL (Robinson et al., 2021). A Z-score < 2 was defined as normal, a Z-score ≥ 2 to < 2.5 as mild dilatation, and a Z-score ≥ 2.5 to < 5 as a small aneurysm. In this study, a Z score greater than 2 was utilized to define the presence of CAL. The left ventricular ejection fraction (LVEF) was assessed using the biplanar Simpson method, employing both the apical four-chamber and apical two-chamber views. This method calculated the change in left ventricular volume by tracing the endocardial borders at end-diastole and end-systole, with the formula: LVEF = [(left ventricular end-diastolic volume - left ventricular end-systolic volume)/left ventricular end-diastolic volume] × 100%. The normal reference value for LVEF was set at ≥ 55% to evaluate left ventricular systolic function.

### 2.3 Serological examination

Fasting venous blood (4 mL) of the patients was collected within 24 hours after admission (on the 5th to 10th day of the disease course, in the acute phase, before IVIG treatment), and the analysis was completed within 8 hours. Levels of immunoglobulins IgG, IgM, and IgA, as well as complement components C3 and C4, were measured using the Siemens BN-II special protein analyzer. Serum levels of CRP, TNF-α, IFN-γ, IL-2, IL-4, IL-6, and IL-10 were quantified using enzyme-linked immunosorbent assay (ELISA), performed strictly according to the reagent manufacturer’s instructions. T lymphocyte subsets (CD4^+^, CD8^+^), B lymphocytes (CD19^+^), NK cells, and regulatory T cells (Treg cells, indirectly assessed via Foxp3 mRNA expression) were identified using flow cytometry or immunomagnetic bead techniques.

### 2.4 Detection of gut microbiota

Fresh fecal samples were collected within 48 hours after admission (on the 5th to 10th day of the disease course, in the acute phase, before IVIG treatment) and were placed in sterile cryogenic tubes, stored at −80°C in the refrigerator. The sampling time for microbiome analysis was earlier than the final diagnosis of CAL (the first ultrasound re-examination in the 2nd to 3rd week of the disease), so the Ace index reflected the gut microbiota status before the diagnosis of CAL, rather than secondary changes after diagnosis. Before collecting the stool samples from all the children, no antibiotics or probiotic preparations were used. The samples were immediately placed in sterile cryovials and stored at −80°C in a refrigerator, with the freezing period not exceeding 6 months. The DNA extraction and library construction were completed in the same batch to reduce batch effects. The diversity of the gut microbiota was analyzed using 16S rDNA high-throughput sequencing technology. PCR amplification was performed in the V3-V4 region, with primers 338F (5’-ACTCCTACGGGAGGCAGCAG-3’) and 806R (5’-GGACTACHVGGGTWTCTAAT-3’).

PE300 sequencing was performed on the DNBSEQ-G400 sequencing platform (BGI, Shenzhen, China). The original sequences were quality-filtered using FastP (v0.23.4), with sequences having an average quality score lower than Q20 and sequences containing ambiguous bases being removed. Chimeric sequences were detected and removed using Vsearch (v2.22.1). After quality control, the number of valid sequences for each sample was ≥ 15,000, and the average sequencing depth was 28,456 sequences per sample. To correct for the sequencing depth differences between samples, all sample sequences were subjected to sparse processing (rarefaction) based on the minimum sample sequence number.

### 2.5 Biological analysis

The OTU sequences were analyzed using the QIIME 2 (version 2023.5) software. Taxonomic annotations for the OTUs were performed based on the Silva database (version 138). Based on the species annotation results, the following indicators were analyzed:

1. α diversity analysis: The Ace index (an α diversity index reflecting the species richness of the gut microbiota) and the Shannon index (an index reflecting the diversity and evenness of the community) were calculated. The differences in inter-group α diversity were compared using the Mann-Whitney U test, and multiple comparisons were corrected for the false discovery rate (FDR) using the Benjamini-Hochberg method.
2. β diversity analysis: The differences in microbial community structures between samples were calculated based on the Bray-Curtis distance. Principal coordinate analysis (PCoA) was used to visualize the β diversity. The differences in β diversity between groups were tested using permutation multivariate analysis of variance (PERMANOVA, with 999 permutations).
3. Species composition analysis: The relative abundance of species in each group of samples was calculated at the phylum and genus levels. The key bacterial genera of concern included *Bifidobacterium*, *Lactobacillus*, *Bacteroides*, *Enterococcus*, and *Escherichia*. The genera with significant differences between groups were tested using the Wilcoxon rank sum test, and the FDR method was used for correction (q < 0.05 was considered statistically significant).

### 2.6 Statistical analysis

Statistical analyses were performed using SPSS version 23.0. The normality of the measurement data was evaluated using the Shapiro-Wilk test. Data that followed a normal distribution were expressed as mean ± standard deviation, and comparisons between the two groups were conducted using the independent sample t-test, Data that did not follow a normal distribution were expressed as median (interquartile range) [M (P25, P75)], and comparisons between the two groups were performed using the Mann-Whitney U test. The Pearson test was used for correlation analysis to screen the variables related to the occurrence of CAL in KD. The variable screening strategy was as follows: variables with P<0.05 in univariate analysis were included in the multivariate model. Based on clinical rationality and literature reports, age, gender, and duration of fever were predetermined as correction variables, and were forcibly included in the model regardless of whether the results of univariate analysis were significant or not. The odds ratio (OR) and 95% confidence interval (95% CI) were calculated.

The Hosmer-Lemeshow test was used to evaluate the goodness of fit of the model. Due to the limited sample size (57 positive cases), the number of independent variables was controlled to be within 9, and stepwise regression was not employed. ROC curve analysis was used to evaluate the discriminatory efficacy of each indicator and the combined detection.

The stability of the combined detection model was evaluated using 5-fold cross-validation. The dataset was randomly divided into 5 equal parts. Each time, 4 parts were used for training the model and 1 part was used for validation. This process was repeated 5 times. A difference was considered statistically significant when P < 0.05. In the analysis of microbial group differences, multiple comparisons were controlled for the false discovery rate (FDR) using the Benjamini-Hochberg method, with q < 0.05 considered statistically significant.

To assess whether the sample size was sufficient to detect the difference in α diversity (Ace index) between the two groups, a *post-hoc* power analysis was conducted in this study. Using the mean Ace index of two groups (404.14 ± 70.48 vs. 464.31 ± 95.92), sample size (57 vs. 102), and α = 0.05 as parameters, an independent sample t-test model was used to compare the means of two samples. The calculated power was 0.87, indicating that the sample size of this study has sufficient statistical confidence (Power ≥ 0.80) in detecting differences in Ace index between the two groups.

## 3 Results

### 3.1 Comparison of general data of the two groups

**Figure 1:**
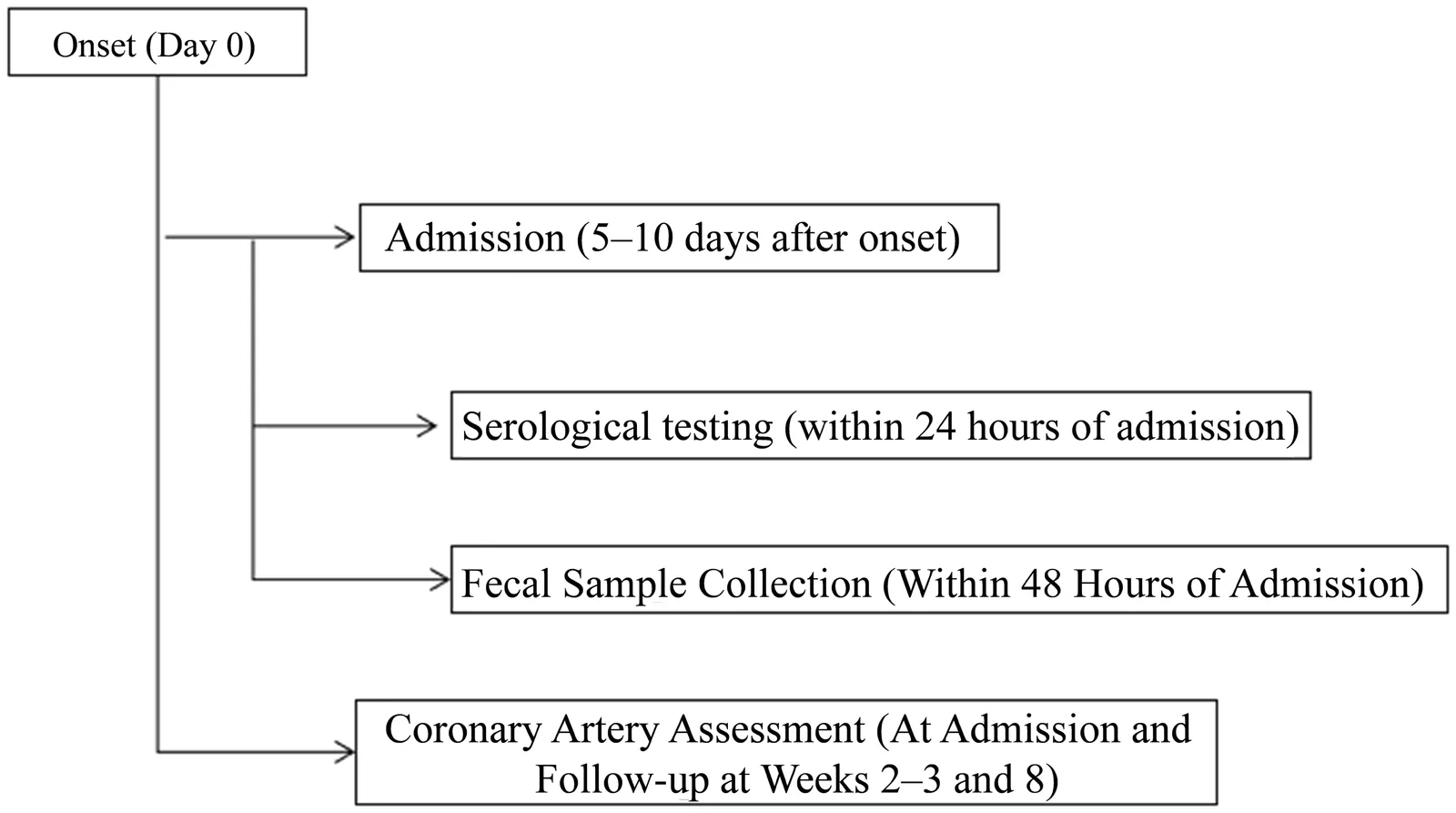
The research process of this study.

The patient’s disease course data, including the onset time and the testing time, were presented in **Figure 1**. No significant differences were observed between the two groups in terms of age, gender, typical clinical symptoms (fever, rash, cervical lymph node enlargement, and strawberry tongue), LVEF and IVIG administration time (*P*>0.05, **Table 1**). The age subgroup analysis showed that there was no significant difference in the proportion of children aged < 3 months, 3 months to 5 years, and > 5 years between the two groups (all *P>0.05*). There was no significant difference in the incidence of gastrointestinal symptoms between the two groups (15 cases (26.32%) in the CAL group vs. 22 cases (21.57%) in the control group, χ² = 0.470, P = 0.493, **Table 1**).

**Table 1:** Comparison of the general data of the two groups.

Characteristics	The CAL group (n=57)	The control group (n=102)	t/χ²	*P*
Age (Year)	2.39 ± 0.76	2.50 ± 0.70	0.891	0.374
Age subgroups			0.085	0.958
< 3 months	2 (3.51)	3 (2.94)		
3 months to 5 years	50 (87.72)	91 (89.22)		
> 5 years	5 (8.77)	8 (7.84)		
Gender (Male/Female)	46/11	72/30	1.204	0.273
Weight (kg)	12.35 ± 2.18	12.68 ± 2.05	0.942	0.348
Fever (Case)	57	100	–	0.102
Rash (Case)	25	55	0.856	0.355
Cervical lymph node enlargement (Case)	42	76	0.012	0.913
Strawberry tongue (Case)	35	74	0.342	0.559
Duration of fever (Day)	7.21 ± 1.83	6.84 ± 1.52	1.367	0.174
IVIG administration time (days of onset)	6.85 ± 1.42	6.63 ± 1.35	0.971	0.333
Duration of rash (Day)	7.52± 1.22	7.32 ± 1.19	1.007	0.315
Duration of cervical lymph node enlargement (Day)	7.11 ± 0.67	6.93 ± 0.91	1.308	0.193
Duration of strawberry tongue (Day)	12.85 ± 2.52	12.64 ± 2.76	0.474	0.636
Duration of conjunctival congestion (Day)	7.83 ± 0.75	7.78 ± 0.91	0.353	0.725
LVEF(%)	62.35 ± 4.52	63.11 ± 3.87	1.117	0.266
Antibiotic use history	8 (14.04)	12 (11.76)	0.169	0.681
Probiotic use history	3 (5.26)	7 (6.86)	0.156	0.693
Gastrointestinal symptoms	15 (26.32)	22 (21.57)	0.470	0.493
Breastfeeding	34 (59.65)	68 (66.67)	0.784	0.376

2.2 Comparison of α diversity, β diversity and species composition of gut microbiota between two groups of pediatric patients.

There was no significant difference in the Shannon index between the control and CAL groups (*P*>0.05). However, the Ace index (α diversity index reflecting the species richness of gut microbiota) was significantly lower in the CAL group compared to the control group (*P* < 0.001, **Table 2**). There was no significant difference in the Shannon index between the two groups (*P > 0.05*, **Table 2**).

**Table 2:** Comparison of gut microbiota α-diversity in pediatric patients (*x* ± *s*).

Group	Cases	Ace index	Shannon index
The CAL group	57	404.14 ± 70.48	2.28 ± 0.97
The control group	102	464.31 ± 95.92	2.32 ± 0.81
*t*		4.149	0.278
*P*		<0.001	0.112

The β-diversity analysis results showed that the Bray-Curtis distance within the CAL group was 0.52 ± 0.18, which was higher than that of the control group (0.45 ± 0.15). It indicated that there was greater microbial heterogeneity among individuals in the CAL group. The PERMANOVA test revealed that there was a statistically significant difference in the microbial community structure between the two groups (F = 2.134, P = 0.028, **Table 3**).

**Table 3:** Comparison of β-diversity of intestinal microbiota and differences between the two groups of children.

Group	Cases	Bray-Curtis distance (within group mean ± standard deviation)	PERMANOVA test
The CAL group	57	0.52 ± 0.18	—
The control group	102	0.45 ± 0.15	—
Statistical value	—	t=2.531	F=2.134
*P*	—	0.012	0.028

At the phylum level, the gut microbiota of both groups of children were mainly composed of *Firmicutes*, *Bacteroidetes*, and *Proteobacteria*. There was no significant difference in the composition of the microbiota between the two groups at the phylum level (*P>0.05*).

At the genus level, the relative abundance of *Bifidobacterium* in the CAL group was lower than that in the control group, while the relative abundance of *Enterococcus* was higher than that in the control group. The differences were statistically significant (*P < 0.05*). There was no significant difference in the genera *Lactobacillus* and *Bacteroides* between the two groups (*P > 0.05*, **Table 4**).

**Table 4:** Comparison of relative abundance of gut microbiota at the genus level between two groups of children (%).

Genus	The CAL group (n=57)	The control group (n=102)	Z value	P value	q value
*Bifidobacterium*	2.34 ± 0.89	3.87 ± 1.12	-3.152	0.002	0.008
*Lactobacillus*	1.85 ± 0.72	2.01 ± 0.68	-0.742	0.458	0.523
*Bacteroides*	28.56 ± 8.34	26.72 ± 9.15	0.835	0.404	0.485
*Enterococcus*	1.56 ± 0.67	0.89 ± 0.34	3.418	0.001	0.004

The q value was the FDR corrected value using the Benjamin Hochberg method. The Wilcoxon rank sum test was used for inter-group comparison.

### 3.2 Comparison of serological indicators between two groups

Additionally, no significant differences were observed in the levels of serum IgG, IgM, IgA, complement C3, complement C4, CD19^+^, and Treg between the two groups (*P*>0.05). Patients in the CAL group exhibited significantly elevated levels of serum CRP, TNF-α, IFN-γ, IL-2, IL-4, IL-6, IL-10, and CD4^+^/CD8^+^ compared to those in the control group (*P* < 0.001, **Table 5**).

**Table 5:** Comparison of serological indicators between two groups (*x* ± *s*).

Group	The CAL group (n=57)	The control group (n=102)	*t/χ^2^*	*P*
IgG (g/L)	6.72 ± 1.22	6.84 ± 1.06	0.648	0.518
IgM (g/L)	1.26 ± 0.28	1.31 ± 0.21	1.274	0.205
IgA (g/L)	0.77 ± 0.25	0.74 ± 0.27	0.690	0.491
Complement C3 (g/L)	1.27 ± 0.45	1.22 ± 0.30	0.838	0.403
Complement C4 (g/L)	0.31 ± 0.10	0.29 ± 0.08	1.380	0.170
CRP (mg/L)	87.79 ± 23.96	68.46 ± 26.85	4.521	<0.001
TNF-α (ng/mL)	27.59 ± 7.89	11.84 ± 4.82	15.626	<0.001
IFN-γ (ng/mL)	38.52 ± 9.63	12.81 ± 3.30	24.556	<0.001
IL-2 (pg/mL)	6.36 ± 2.26	3.67 ± 1.25	9.675	<0.001
IL-6 (pg/mL)	380.23 ± 70.24	290.27 ± 41.80	10.130	<0.001
IL-10 (pg/mL)	58.07 ± 15.32	20.52 ± 5.17	22.604	<0.001
IL-4 (pg/mL)	5.96 ± 1.77	4.12 ± 0.92	8.631	<0.001
CD4^+^/CD8^+^	4.15 ± 1.14	2.41 ± 0.25	14.824	<0.001
CD19^+^ (%)	23.69 ± 5.75	24.14 ± 3.78	0.594	0.553
NK (%)	10.85 ± 3.74	11.62 ± 3.41	1.319	0.189
Treg (%)	6.02 ± 2.27	5.77 ± 1.82	0.759	0.449

### 3.3 Comparison of serological indicators and Ace index in children with different degrees of KD

The children were divided into the mild/moderate group (IVIG-sensitive) and the severe group (IVIG-resistant) according to the severity of the disease. The differences in Ace index and serological indicators between the two groups were compared. The results showed that the Ace index in the severe group was lower than that in the mild/moderate group, while CRP, TNF-α, IFN-γ, IL-6, IL-10, IL-4, and CD4^+^/CD8^+^ were all higher in the severe group than in the mild/moderate group (all *P < 0.05*, **Table 6**).

**Table 6:** Comparison of Ace index and serological indicators between the mild/moderate group and the severe group.

Indicators	The mild/moderate group (n=138)	The severe group (n=21)	t value	P vaule
Ace index	452.67 ± 91.23	378.45 ± 68.72	3.562	<0.001
CRP (mg/L)	72.38 ± 25.64	95.27 ± 28.13	3.810	<0.001
TNF-α (ng/mL)	16.35 ± 6.28	31.42 ± 8.15	10.128	<0.001
IFN-γ (ng/mL)	20.17 ± 5.24	40.68 ± 9.72	14.352	<0.001
IL-6 (pg/mL)	310.52 ± 42.37	398.64 ± 68.25	7.892	<0.001
IL-10 (pg/mL)	30.48 ± 8.52	62.35 ± 14.28	13.624	<0.001
IIL-4 (pg/mL)	4.68 ± 1.02	6.52 ± 1.85	7.104	<0.001
CD4^+^/CD8^+^	2.85 ± 0.52	4.52 ± 1.08	11.356	<0.001

### 3.4 The correlation between gut microbiota with clinical data and serological indicators

Pearson correlation analysis demonstrated that the Ace index had no significant correlation with the clinical data (*P*>0.05). However, the Ace index was found to be significantly negatively correlated with serum levels of CRP, TNF-α, IFN-γ, IL-2, IL-4, IL-6, IL-10, and CD4^+^/CD8^+^ (*P* < 0.05). In contrast, no significant correlation was observed between the Shannon index and either clinical data or serological indicators (*P*>0.05, **Table 7**). These findings suggested that alterations in the abundance of gut microbiota might be directly associated with the inflammatory state of the disease, independent of demographic characteristics and clinical phenotypes. The Shannon index had no correlation with the above indicators. This further supported that the abundance of gut microbiota, rather than the evenness, was involved in the pathological process of CAL.

**Table 7:** The correlation between gut microbiota with clinical data and serological indicators.

Indicators	Ace index		Shannon index	
	*r*	*P*	*r*	*P*
Age	-0.085	>0.05	0.124	>0.05
Gender	0.112	>0.05	-0.092	>0.05
Typical clinical symptoms	0.155	>0.05	0.075	>0.05
CRP	-0.428	<0.05	0.181	>0.05
TNF-α	-0.519	<0.05	0.169	>0.05
IFN-γ	-0.483	<0.05	0.216	>0.05
IL-2	-0.464	<0.05	0.193	>0.05
IL-6	-0.396	<0.05	0.147	>0.05
IL-10	-0.372	<0.05	0.112	>0.05
IL-4	-0.435	<0.05	0.174	>0.05
CD4^+^/CD8^+^	-0.523	<0.05	0.235	>0.05

### 3.5 Multivariate logistic regression analysis of KD complicated with CAL (screening of associated factors)

**Figure 2:**
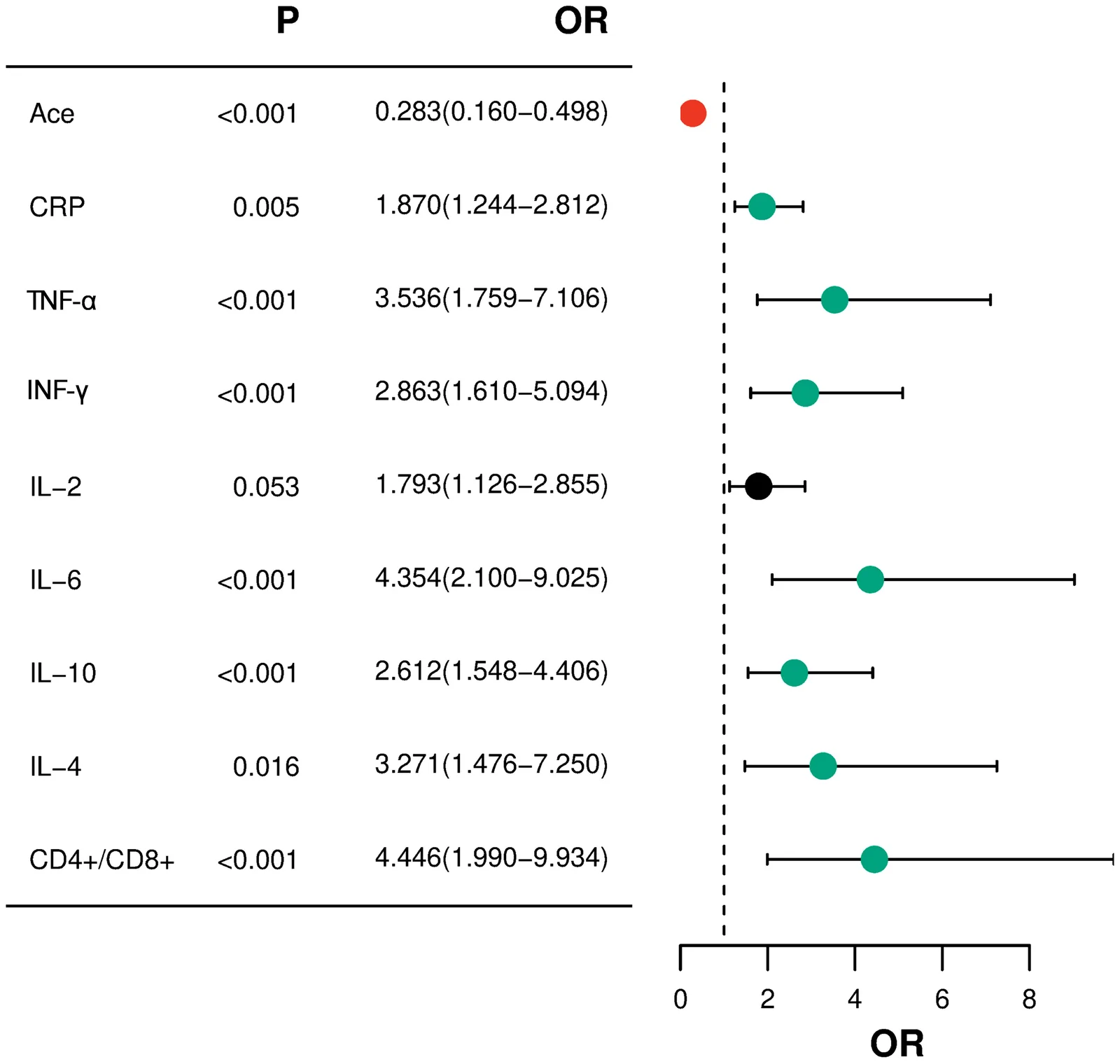
Forest plot of the multivariate logistic regression analysis of the main exposure factors for KD complicated with CAL. The figure showed the OR value and 95% confidence interval (CI) of Ace index, CRP, TNF-α and other indicators. The results showed that except for the Ace index (OR < 1, which was a protective factor), all the other indicators were independent risk factors (*P* < 0.05). The OR value of CD4^+^/CD8^+^ was the highest (4.446, 95%CI 1.990-9.934), suggesting that the imbalance of T lymphocyte subsets was closely related to the increased risk of coronary artery lesions.

After adjusting for age, gender and duration of fever, multivariate Logistic regression analysis showed that the Ace index, pro-inflammatory factors (CRP, TNF-α, IFN-γ, IL-6, IL-4), and the CD4^+^/CD8^+^ ratio were independently associated with concurrent CAL, and the anti-inflammatory factor IL-10 was also independently associated with CAL (*P < 0.05*, **Table 8** and **Figure 2**). Variable selection was based on the following criteria: variables with a P value less than 0.05 in the univariate analysis were included in the multivariate model. In combination with literature reports and clinical rationality, age, gender, and duration of fever were pre-emptively included as correction variables. The goodness-of-fit test of the model (Hosmer-Lemeshow test) yielded a P value of 0.326, indicating that the model fits well. After adjusting for age, gender, and duration of fever, the above variables were all P<0.05 and still independently associated with CAL. Among the correction variables, age (OR = 1.021, 95% CI 0.923 - 1.130, P = 0.690), gender (β = 0.301, P = 0.443), and duration of fever (OR = 1.083, 95% CI 0.858 - 1.367, P = 0.519) all had no statistical significance in this model (**Table 8**).

**Table 8:** Multivariate logistic regression analysis of KD complicated with CAL (Screening of associated factors).

Indicators	β value	SE	*Wald* value	*P* value	OR	95%*CI*
Main exposure factors						
Ace index	-1.265	0.284	20.252	<0.001	0.283	0.160-0.498
CRP	0.626	0.208	9.058	0.005	1.870	1.244-2.812
TNF-α	1.263	0.356	12.587	<0.001	3.536	1.759-7.106
IFN-γ	1.052	0.294	12.804	<0.001	2.863	1.610-5.094
IL-2	0.588	0.304	3.742	0.053	1.800	0.992-3.266
IL-6	1.471	0.372	15.636	<0.001	4.354	2.100-9.025
IL-10	0.960	0.267	12.928	<0.001	2.612	1.548-4.406
IL-4	1.185	0.406	8.519	0.016	3.271	1.476-7.250
CD4^+^/CD8^+^	1.492	0.410	13.242	<0.001	4.446	1.990-9.934
Correction variables						
Age (year)	0.021	0.052	0.159	0.690	1.021	0.923-1.130
Gender (Male=1, Female=0)	0.301	0.385	0.611	0.443	—	—
Duration of fever (d)	0.080	0.118	0.456	0.519	1.083	0.858-1.367

The Hosmer-Lemeshow test P = 0.326 indicated that the model fits well. Gender was a binary variable (female = 0, male = 1), and the OR value was not applicable. The correction effect was reflected by the β coefficient and P value. Although the correction variables were included in the model, they were not the exposure factors of this study. The statistical values of these variables were listed in the table for reference only.

### 3.6 The discriminatory efficacy of gut microbiota and serum indicators for KD complicated with CAL

**Figure 3:**
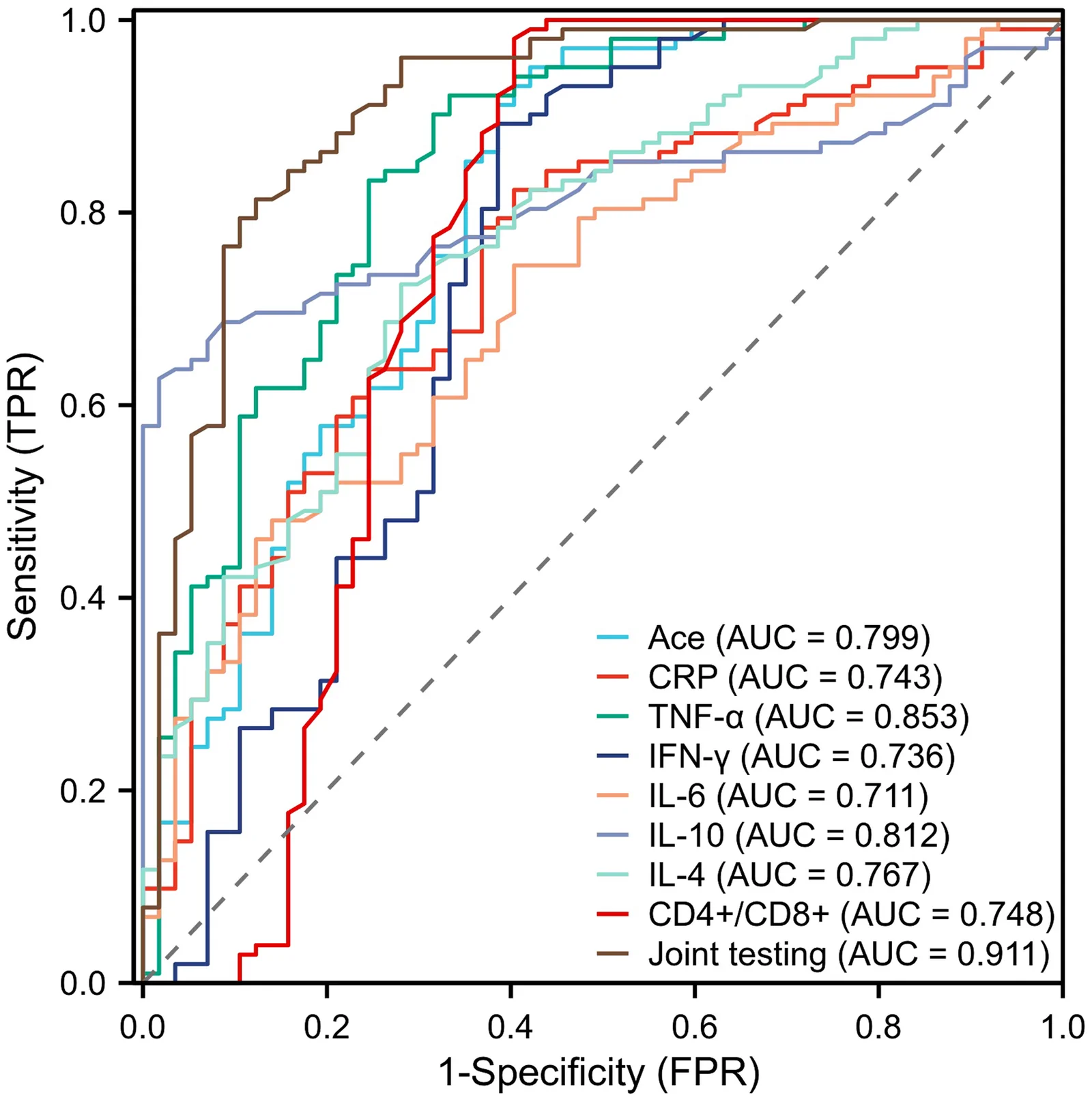
Prediction ROC curve of gut microbiota and serum indicators for KD complicated with CAL. The figure showed the ROC curves of Ace index, CRP, TNF-α and other single indicators and combined detection. The AUC of the combined detection was 0.911 (95%CI 0.860-0.962), the sensitivity was 87.72%, the specificity was 81.37%, and the Youden index was 0.691, suggesting that the combined detection could effectively distinguish the coronary artery lesion group from the non-coronary artery lesion group.

**Figure 4:**
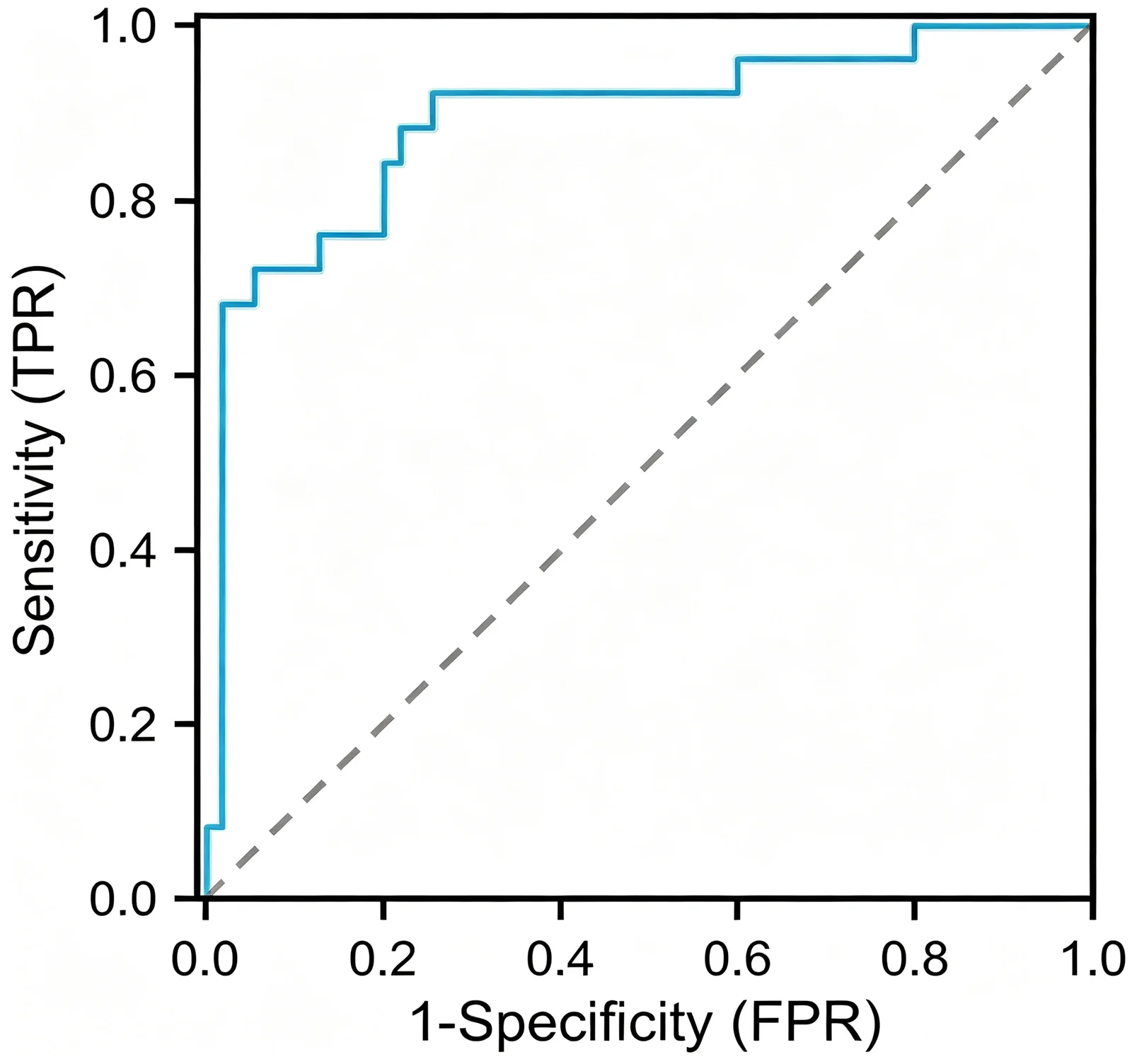
ROC curves of each fold in 5-fold cross-validation. The average AUC was 0.891 (95% CI 0.84 - 0.94), which was close to the AUC of the training set (0.911), suggesting that the model had good stability and generalization ability. The diagonal (dotted line) represented the opportunity line where AUC = 0.5. The 5-fold cross-validation method: The dataset was randomly divided into 5 equal parts. Each time, 4 parts were used for training the model and 1 part for validation. This process was repeated 5 times, and the average AUC was taken as the evaluation index for the stability of the model.

ROC curve analysis demonstrated that the AUC of the combined detection of the Ace index, CRP, TNF-α, IFN-γ, IL-6, IL-10, IL-4, and CD4^+^/CD8^+^ ratio was 0.911, with specificity and sensitivity values of 87.72% and 81.37%, respectively (**Table 9** and **Figure 3**). This indicated a high discriminatory efficacy for KD complicated with CAL. The average AUC of 5-fold cross-validation was 0.891 (95% CI 0.84 - 0.94), which was close to the AUC of the training set (0.911, **Figure 4**).

**Table 9:** The discriminatory power of gut microbiota and serum indicators for KD complicated with CAL.

Indicators	AUC	Specificity	Sensitivity	Youden’s index	Cut-off value	*P* value	95% *CI*
Ace index	0.799	57.90	95.10	0.530	421.75	<0.05	0.721-0.876
CRP	0.743	59.65	82.35	0.420	73.30 mg/L	<0.05	0.663-0.823
TNF-α	0.853	66.67	92.16	0.588	24.85 ng/mL	<0.05	0.787-0.918
IFN-γ	0.736	61.40	89.22	0.506	11.60 ng/mL	<0.05	0.643-0.829
IL-6	0.711	59.65	74.51	0.342	327.50 pg/mL	<0.05	0.629-0.793
IL-10	0.812	98.25	62.75	0.610	25.20 pg/mL	<0.05	0.746-0.878
IL-4	0.767	71.93	72.55	0.445	5.15 pg/mL	<0.05	0.691-0.843
CD4^+^/CD8^+^	0.748	59.65	98.04	0.577	3.42	<0.05	0.649-0.848
Combined detection	0.911	87.72	81.37	0.691	——	<0.05	0.860-0.962

The Youden index reflects the overall efficacy of the diagnostic test. Its value is the sum of the sensitivity and specificity minus 1. The Youden index of the combined test was 0.691, which was higher than that of a single indicator, indicating that the combined test can better distinguish the coronary artery lesion group from the control group. The above results were a retrospective analysis and cannot be regarded as direct evidence for a predictive model. Further verification is needed through a prospective study.

## 4 Discussion

Kawasaki disease (KD) is a prevalent autoimmune inflammatory disorder in children. It primarily affects small and medium-sized blood vessels, particularly the coronary arteries, and may result in coronary artery dilation, stenosis, and myocardial infarction (Han et al., 2024). Currently, CAL caused by KD is recognized as the most prevalent acquired heart disease among children in developed nations and may also be one of the risk factors for ischemic heart disease in adulthood (Qian et al., 2020). This study sought to investigate potential biomarkers for CAL by examining the diversity of gut microbiota and serum inflammatory indicators in children with KD, thereby providing a novel foundation for the early prediction of CAL.

In recent years, numerous studies have shown that gut microbiota and its metabolites may play a role in the pathogenesis and progression of cardiovascular diseases. Factors such as the chronic inflammatory state of cardiovascular disease, immune dysfunction, and pharmacological interventions (e.g., antibiotics and immunomodulators) may disrupt the balance of intestinal flora. Such dysbiosis is associated with intestinal inflammation and reduced intestinal barrier integrity. In contrast, this process increases the circulation levels of bacterial structural components and microbial metabolites, thereby facilitating the progression of cardiovascular disease (Jin et al., 2019; Rahman et al., 2022). Researches have demonstrated that the composition of intestinal flora underwent changes during the acute phase of KD. This can result in a reduction of metabolites such as short-chain fatty acids, damaging the integrity of the intestinal barrier, and facilitating the release of proinflammatory substances like lipopolysaccharide into the bloodstream, thereby activating a systemic inflammatory cascade reaction (Esposito et al., 2019; Yu et al., 2025). Furthermore, studies suggest that superantigens associated with KD may partially originate from the intestinal flora, and their promoters are likely to have invaded from the human intestinal tract (Kaneko et al., 2020). The imbalance in the proportion of gut microbiota may lead to a decrease in beneficial metabolites, such as short-chain fatty acids (SCFAs). While it also promotes the excessive proliferation of Gram-negative bacteria and the subsequent release of lipopolysaccharide (LPS). When the integrity of the intestinal barrier is compromised due to inflammation, LPS can pass through the intestinal mucosa and enter the bloodstream, subsequently activating the Toll-like receptor 4 (TLR4)/NF-κB signaling pathway within the circulatory system. This activation induces the substantial release of pro-inflammatory cytokines such as TNF-α and IL-6, thereby exacerbating the systemic vascular inflammatory response (Sun and Xing, 2021; Li and Xing, 2023). In the present study, the Ace index of children with CAL was decreased, indicating a reduction in the abundance of intestinal microbiota. This reduction was characterized by a lower proportion of beneficial bacteria, such as *Bifidobacterium* and *Lactobacillus*, and a relative increase in opportunistic pathogens. It possibly accompanied by an increase in the blood load of LPS derived from Gram-negative bacteria. These findings were corroborated by elevated inflammatory indicators such as serum CRP and TNF-α. In children diagnosed with KD, gastrointestinal symptoms are frequently observed, such as abdominal pain, diarrhea, vomiting, gallbladder hydronephrosis, jaundice, abdominal distension, paralytic intestinal obstruction, and pancreatitis. These gastrointestinal symptoms have been proven to be related to the severity of KD (Lazea et al., 2019). A study indicated that compared to healthy children, the abundances of bacterial such as *Lactobacillus* and *Veillonella* in children with KD were significantly reduced, while the abundance of bacteria such as *Bacteroides* and *Enterococcus* were significantly increased (Teramoto et al., 2023). Similar to these research results, the present study confirmed a significant decrease in the Ace index among children with CAL, whereas the Shannon index showed no significant change. The relative abundances of beneficial bacteria such as *Bifidobacterium* and *Lactobacillus* were decreased, while the relative abundances of opportunistic pathogens such as *Enterococcus*, *Clostridium*, and *Escherichia* were increased. This suggested a reduction in the species richness of the intestinal microbiota in children with CAL, primarily due to the depletion of beneficial bacteria, while the evenness of species distribution was not significantly affected. The Ace index is indicative of the species richness of the microbiota, representing the total number of species. In contrast, the Shannon index accounts for both species richness and evenness, providing a more comprehensive reflection of the diversity within the microbiota. This suggests that certain pathogenic or opportunistic bacteria may proliferate in the CAL group, resulting in a disruption of the microbial structure. Subsequently it facilitate the development of CAL through immune activation and inflammatory responses (Zeng et al., 2023). Furthermore, dysbiosis of the gut microbiota can impair nutrient absorption and cause metabolic abnormalities, which may adversely affect the cardiovascular system (Lippi et al., 2017). Lazea et al. (2019) found that the gastrointestinal manifestations of children with KD were related to the severity of the disease. In this study, the incidence of gastrointestinal symptoms in the CAL group was 26.32%, while that in the control group was 21.57%. There was no significant difference between the two groups (P = 0.493). The Pearson correlation analysis showed that the Ace index was not significantly correlated with the typical clinical manifestations of KD (fever, rash, cervical lymphadenopathy, strawberry tongue) (P > 0.05). The above results suggested that in the study population, the association between the reduction in gut microbiota richness (Ace index) and CAL may be independent of gastrointestinal symptoms and typical clinical manifestations. That is to say, the impact of gut microbiota imbalance on CAL may not depend on the severity of clinical symptoms, but rather act directly through inflammatory immune pathways. However, due to the limitation of sample size, this study did not conduct subgroup analysis based on the presence or absence of gastrointestinal symptoms. The above conclusions require further research for verification. Additionally, the microbial group samples in this study were collected within 48 hours after admission (on the 5th to 10th day of the onset), and the final diagnosis of CAL was based on the echocardiogram re-examination results in the 2nd to 3rd week of the disease course. Therefore, the decrease in the Ace index occurred before the diagnosis of CAL, rather than as a secondary change following the diagnosis. This temporal sequence suggests that the reduction in the richness of the gut microbiota may be involved in the early pathological process of CAL, rather than merely reflecting the inflammatory state caused by established coronary artery damage. However, samples were not collected earlier in the onset of the disease (such as within 3 days of onset) in this study, and the influence of disease progression on the Ace index cannot be completely ruled out. Therefore, the Ace index should be considered as a disease severity marker associated with CAL, rather than an independent predictor.

KD is an autoimmune disorder. In patients with this condition, the immune system becomes abnormally activated, attacking the vascular endothelial and smooth muscle cells, resulting in damage to the vascular walls and triggering inflammatory responses. This may lead to relaxation and dilation of the vascular walls (Wang and Li, 2022). During the acute phase of KD, the immune system releases a substantial amount of inflammatory cytokines, such as interleukin and TNF-α. These cytokines not only intensify the systemic inflammatory response but also may directly damage the endothelial cells of the coronary arteries, leading to CAL (Noval Rivas and Arditi, 2023). This study identified significant abnormalities in serum CRP, TNF-α, IFN-γ, IL-6, IL-10, IL-4, and CD4^+^/CD8^+^ indicators in children with concurrent CAL, suggesting all of them were independently associated with the occurrence of KD complicated by CAL. Serum CRP, TNF-α, IFN-γ, and other cytokines are routinely utilized in clinical diagnostics for various diseases. Consistent with the findings of this study, previous research has demonstrated that during the acute phase of KD, particularly in patients with concurrent cardiovascular disease, there is a marked elevation in inflammatory markers such as serum CRP, white blood cell count, and lymphocyte count (Shuai et al., 2023; Alkan et al., 2024). Another study reported that the serum TNF-α level of children with KD was significantly higher than that of healthy controls. TNF-α is known to stimulate the activation of endothelial cell adhesion molecules, compromise vascular integrity, and influence proliferation of endothelial cell and repair processes (Chen et al., 2023). Serum IL-6 and CRP are widely recognized as inflammatory markers. It has been reported that IL-6 changes earlier than CRP during inflammatory responses. Consequently, IL-6 is a crucial biomarker for assessing patients’ disease status and reflecting their immune system function (Li et al., 2024). Nevertheless, no significant differences were observed in humoral immune indicators, such as IgG, IgM, IgA, and complement components C3 and C4. This might suggest that the immunopathological mechanism of KD complicated by CAL is more dependent on the T cell-mediated inflammatory response rather than the antibody-mediated humoral immune response (Wang et al., 2025). Additionally, during the acute phase of KD, tissue cells, including macrophages and dendritic cells, are activated and secrete large amounts of cytokines such as TNF-α and IL-6, thereby exacerbating the vascular inflammatory response. This may be an important reason for the differences in cytokine levels between the two groups (Li et al., 2019). IL-1 is a significant pro-inflammatory factor in innate immunity, contributing to the early stages of inflammation via the NLRP3 inflammasome pathway. However, its role in the pathway acquired immunity, particularly in T cell-mediated inflammation is relatively upstream and indirect (Wang Y. et al., 2023). In this study, the Ace index still showed an independent association with CAL after multivariate correction, suggesting a statistical association between the reduction in gut microbiota richness and CAL. This study did not measure serum LPS or zonulin levels, thus unable to verify whether changes in intestinal permeability or the LPS/TLR4/NF-κB pathway were involved. The correlation between the Ace index and systemic cytokines only reflects a statistical association and cannot infer a causal relationship. Previous studies have confirmed that the Ace index is an independent influencing factor for CAL in children with KD (Tao and Lang, 2024). The Ace index, as a distant indicator of intestinal microecological imbalance, has a statistically significant association with CAL. However, the specific mechanism requires further research and verification. Li et al. (2024) demonstrated that indicators such as CRP, TNF-α, IFN-γ, IL-6, IL-10, IL-4, and CD4^+^/CD8^+^ are associated with the severity of KD. This study further divided the children into mild/moderate group and severe group based on whether they were resistant to IVIG. The results showed that the levels of the above indicators in the severe group were consistent with the results of the CAL group in terms of direction, and the Ace index was also decreased. This suggested that the differences in the various indicators observed in this study may partially reflect the overall severity of the disease rather than being completely specific to the CAL itself. However, after adjusting for disease severity (IVIG resistance), multiple regression analysis showed that Ace index and the above serological indicators were still independently associated with CAL (*P<0.05*). This suggested that the association between the above indicators and CAL may not be fully explained by disease severity, and there may be other specific mechanisms. Due to sample size limitations (only 21 cases in the severe group), the analysis results of this subgroup need to be interpreted with caution and further validated through large sample studies.

In KD, the dysregulation of T lymphocyte subsets may result in excessive activation of immune system. It leads to an autoimmune attack on vascular endothelial and smooth muscle cells, thereby causing systemic vasculitis (Lo et al., 2023). Evidence indicates that children with KD often exhibit an imbalance in T lymphocyte subsets during the acute phase, with an increased CD4^+^/CD8^+^ ratio. It may intensify inflammatory responses and contribute to vascular wall damage and CAL (Wu et al., 2021). Furthermore, research has demonstrated that when KD occurs, there is an imbalance in T lymphocyte subgroups and an increase in lymphokine secretion. This can promote the secretion of related antibodies by B lymphocytes and their combination with antigens to deposit in the vascular walls, thereby increasing the risk of vasculitis. The immune system is integral to the pathogenesis and progression of KD complicated by CAL (Xu et al., 2024). The findings of this study suggest that the CD4^+^/CD8^+^ ratio is associated with the development of CAL in KD. An elevated CD4^+^/CD8^+^ ratio causes the immune system of the body to be in an activated state. The lymphokines secreted by CD4^+^ cells increase, promoting the polyclonal activation, proliferation and differentiation of B cells into plasma cells, resulting in an increase in serum immunoglobulins. Additionally, these lymphokines and active mediators may induce the expression of neoantigens in endothelial cells and enhance the secretion of autoantibodies by B cells, thereby causing cytotoxicity of endothelial cells and endothelial cell damage, which leads to vasculitis (Tao et al., 2023). This process is intricately linked to the pathogenesis of KD complicated by CAL. Furthermore, this study found no significant difference in the percentage of regulatory T cells (Treg) between the two groups. The possible reason is that functional changes rather than alterations in the number of Treg cells. This functional impairment could lead to a loss of their immunosuppressive capacity, thereby failing to effectively control the inflammatory response (Guo et al., 2015). Furthermore, although no differences were observed in humoral immune parameters, T-cell subset imbalance and cytokine storm remain central mechanisms in KD complicated with CAL. In this study, the OR value of the CD4^+^/CD8^+^ ratio (4.446) was higher than that of CRP (1.870), suggesting that the imbalance of T cell subsets might be at a more upstream regulatory position in CAL. The vasculitis of KD is centered on T cell-mediated immune damage, and pathological examination confirmed that there was a large amount of CD8^+^ T cell transmural infiltration in the coronary artery wall (Brown et al., 2021). An increase in the CD4^+^/CD8^+^ ratio reflects the imbalance of a relative predominance of helper T cells and a relative deficiency of cytotoxic T cells. And it can lead to an increase in the secretion of lymphokines by CD4^+^ T cells, promote B cell activation and the production of autoantibodies, and induce endothelial cell damage (Fan et al., 2025). Inflammatory markers such as CRP mainly reflect the ultimate effects of inflammation, while the CD4^+^/CD8^+^ ratio directly reflects the core aspect of immune regulation. Therefore, they exhibit a higher effect size in the statistical model.

Furthermore, the results of this study revealed that the Ace index was negatively correlated with CRP, TNF-α, IFN-γ, IL-6, IL-4, and the CD4^+^/CD8^+^ ratio. This indicated that the richness of the gut microbiota has a general stabilizing effect on the immune system of children with KD. A decrease in the richness of the microbiota can lead to a reduction in short-chain fatty acids, damage to the intestinal barrier, and the activation of the TLR4/NF-κB pathway by LPS entering the bloodstream, thereby inducing the release of various pro-inflammatory factors (Bai et al., 2025). The wide negative correlation between the Ace index and a variety of pro-inflammatory markers reflects the extensive regulatory role of the gut microbiota through the immune-inflammatory network on the systemic inflammatory state (Tao and Lang, 2024). There is a significant correlation between the Ace index and the CD4^+^/CD8^+^ ratio. The underlying mechanism may be related to the regulation of T cell differentiation by the gut microbiota (Niewiem and Grzybowska-Chlebowczyk, 2022). The excessive inflammation during the acute phase of KD is mediated by the imbalance between the enhanced Th17/Th1 response and the weakened Th2/Treg response. The reduction in microbiota richness leads to a decrease in short-chain fatty acids, and short-chain fatty acids have the immunomodulatory effect of promoting Treg differentiation and inhibiting Th17 differentiation (Smith et al., 2013; Niewiem and Grzybowska-Chlebowczyk, 2022). Gut microbiota imbalance may activate dendritic cells through the TLR4/NF-κB pathway, promoting Th17 differentiation (Smith et al., 2013). IL-17 has a relatively high OR value in CAL of KD, suggesting that the Th17 pathway is an important direction worthy of further research. The Ace index is a widely used to evaluate gut microbiota diversity. In this study, the combined application of the Ace index and serological indicators may elucidate the complex relationship between gut microbiota dysbiosis and KD, along with its cardiovascular complications. Alterations in gut microbiota diversity may influence immune system function, thereby increasing the risk of KD and its cardiovascular complications. By jointly assessing the Ace index and multiple serological indicators, it is possible to more effectively predict the risk of KD complicated by CAL. This combined detection method may enhance the accuracy and reliability of the prediction, helping to identify high-risk patients and taking appropriate intervention measures.

From a cardiac pharmacology standpoint, the association found in this study suggests the following potential research directions: Given the association between Ace index and CAL, prospective studies can be designed in the future to explore the effect of probiotics (such as bifidobacteria) regulating gut microbiota abundance on vasculitis. The elevated OR values of pro-inflammatory factors such as TNF-α and IL-6 suggest that the use of biological agents, including tocilizumab, can be considered to block the inflammatory cascade reaction. This is particularly applicable to pediatric patients exhibiting elevated Ace index and cytokine levels. The CD4^+^/CD8^+^ imbalance suggests that T cell subsets can be regulated by aspirin combined with immunoglobulin. In the future, the inhibitory effect of JAK inhibitors on T cell activation can be explored. Recent studies based on a limited number of small samples have confirmed that tocilizumab (an IL-6 receptor antagonist) shows potential in controlling systemic inflammation in IVIG-resistant KD. A retrospective analysis of 5 patients with IVIG-resistant KD showed that inflammatory markers in the patients were decreased rapidly after tocilizumab treatment, and no new CAL were observed (Ling et al., 2024). Another case report involving 2 cases of IVIG-resistant KD also supports that tocilizumab can be a potential treatment option for IVIG-resistant KD (Wu et al., 2026). However, the existing evidence mainly comes from small sample case series and lacks large-scale randomized controlled trials to confirm this. Therefore, the application of tocilizumab in KD still requires further research and verification. The samples in this study were all collected during the acute phase of KD (from the 5th to the 10th day of the disease course, before IVIG treatment). This design can reduce the confounding effect of differences in disease progression stages on the indicators, but it cannot assess the dynamic changes of gut microbiota and serological indicators in different stages of the disease (acute phase, subacute phase, and recovery phase). Therefore, this study reflects the cross-sectional association between these indicators and CAL in the acute phase, and cannot determine the causal relationship.

Overall, this study found that the levels of serum CRP, TNF-α, IFN-γ, IL-6, IL-10, IL-4, and the CD4^+^/CD8^+^ ratio were significantly increased in children with KD complicated by CAL, while the Ace index was significantly decreased. There was an independent association between these findings and KD complicated by CAL. The combined detection of various indicators showed good discriminatory ability in the retrospective cohort, but its value as an independent predictor needs to be further verified through prospective studies. This study has certain limitations: (1) This study was a retrospective design. Blood and fecal samples were collected after the children were admitted to the hospital (on the 5th to 10th day of the disease course), and the assessment of CAL was conducted during the clinical course. The time points for detecting biomarkers and CAL were not in a strictly sequential order. Therefore, the analysis results of this study reflected the concurrent correlation between the gut microbiota and serological indicators and CAL. However, it cannot determine the causal relationship between the two, nor to support their use as independent predictors of CAL. (2) The multivariate model of this study only included a limited number of confounding factors. It failed to adequately control for key factors that might affect the composition of the gut microbiota, such as antibiotic exposure, probiotic use, dietary patterns, breastfeeding history, gastrointestinal symptoms, fecal characteristics, sample preservation methods, DNA extraction methods, sequencing depth, and batch effects. These confounding factors may interfere with the observation results of microbial group indicators like the Ace index. (3) The microbiological analysis in this study was limited to 16S rDNA sequencing, with insufficient species-level resolution, and no penalized regression or dimensionality reduction methods were used. Internal validation was not conducted, and the ratio of independent variables to positive events was approximately 1:6, indicating a risk of overfitting.

## Data Availability

The datasets presented in this study can be found in online repositories. The names of the repository/repositories and accession number(s) can be found in the article/supplementary material.
